# Collaborating while competing? The sustainability of community-based integrated care initiatives through a health partnership

**DOI:** 10.1186/1472-6963-6-37

**Published:** 2006-03-20

**Authors:** Thomas Plochg, Diana MJ Delnoij, Nelleke PC Hoogedoorn, Niek S Klazinga

**Affiliations:** 1 Department of Social Medicine, Academic Medical Centre, University of Amsterdam, PO Box 22660, 1100 DD, Amsterdam, The Netherlands; 2 NIVEL Netherlands Institute of Health Services Research, PO Box 1568, 3500 BN, Utrecht, The Netherlands; 3 Verenigde Amstelhuizen, PO Box 1103, 1000 BC Amsterdam, The Netherlands

## Abstract

**Background:**

To improve health-care delivery, care providers must base their services on community health needs and create a seamless continuum of care in which these needs can be met. Though, it is not obvious that providers apply this vision. Experiments with regulated competition in the health systems of many industrialized countries trigger providers to optimize individual organizational goals rather than improve population health from a community perspective. Thus, a tension exists between the need to collaborate and the need to compete. Despite or because of this tension, community health partnerships are being promoted, and this should enforce a needs-based and integrated care delivery.

**Methods:**

In this single case study, we retrospectively explored how local health-care providers in Amsterdam collaborated for more than 30 years, interacting with the changes to the national health-care system. In-depth analysis of interviews, documents and literature focused on the complex relationship between the activities of this health partnership, its nature and its changing context.

**Results:**

The findings revealed that the partnership itself was successful and sustainable over time, although the partnership lost its initial broad explorative nature and narrowed its strategic focus towards care of the elderly. Furthermore, the realized projects – although they enforced integrated care – lost their community-based character. This declining scope of community-based integrated care seems to have been influenced by the incremental introduction of regulated competition in Dutch health care. This casts doubts on the ability of health partnerships to apply a vision of community-based integrated care within the context of competition.

**Conclusion:**

Collaborating health-care providers can build seamless continuums of care in a competitive environment, although these will not automatically maximize community health with limited resources. Active policies with regard to health system design, incentive structures and population-based performance measures are warranted in order to insure that community-based integrated care through health partnerships will be more than just policy rhetoric.

## Background

To improve performance in health care, providers should target their services to the health needs, beliefs and values of the populations they are intended to serve. This orientation enables them to offer an appropriate set of services that maximizes population health given the available resources [1-3]. According to this logic, providers must collaborate and integrate their services, as most health needs cannot be met by any single provider working alone [4-7]. This calls for integration of public health functions, medical care functions and social services at local and regional levels. This has been articulated in the strategic vision of 'community-based integrated care', and features two components [8]: 1) Clear goals must be defined and prioritized within the context of limited resources and based on the population's health needs, beliefs and values; and 2) seamless continuums of care must be built within which the defined goals can best be met. We assume that when health-care providers adopt this vision, population health outcomes within the context of limited resources can be maximized.

In practice, it is not self-evident that health-care providers will join collaborative programmes aimed at improving community health within the context of limited resources. These programmes may imply a loss of power, control or income for individual providers, because they realign health-care delivery in favour of more preventive, social and primary care services [9]. Notwithstanding, large differences exist among industrialized countries, and governments are increasingly experimenting with market mechanisms to encourage competition, which drives down costs [10,11]. In a competitive environment, individual providers have the incentive to maintain their own economic viability and achieve individual organizational goals rather than to take collective action that improves population health. There are often insufficient adjustments made for this behaviour, because most governments fail to systematically embed population health considerations into their health systems and incentive structures [12,13]. Thus, a tension exists between the need to collaborate and the need to compete.

Despite or perhaps because of this tension, health partnerships are being developed and promoted in various countries such as the United Kingdom [14,15], the United States [16,17], and the Netherlands [18-20]. We define a health partnership as a local coalition of independent public health, health care and social care providers that focus on improving community health within the context of limited resources and coordinating an integrated provision of care. Put differently, health-care providers themselves are considered to be collaborating on a voluntary basis, and to be optimizing their collective contribution to population health.

However, there is limited empirical evidence that health partnerships can be effective in this way [21-24]. Moreover, there is little evidence on how to build and sustain health partnerships in the first place [25]. In general, although there is knowledge on which factors are relevant, how these interact and can be managed to create effective health partnerships remains a field of inquiry. The lack of evidence is attributed to the sparse research with a significant time horizon. In order to detect measurable effects, you need to track partnerships and their activities for considerable period of time [5,21,23,25]. Also, the numerous factors influencing partnerships and their activities cannot be effectively isolated from each other and from the wider operating context in which the partnership functions [26]. In conclusion, there is no best way to implement a partnership that improves population health, nor is there one true way of evaluating its successes [21].

In this paper, we present a single case study in which the aforementioned topic has been addressed. We studied how health-care providers in 'South-eastern Amsterdam' (Amsterdam Zuidoost) have been collaborating for more than 30 years. The partnership known as the SGZ (Stichting Gezondheidszorg Zuidoostlob, 'Health-care Foundation for the South-eastern District') and later the Zizo (Zorgintegratie Zuidoost, 'Health-care Integration for the South-east') was founded in 1973 with the aim of shaping the local health system in this urban district, which at that time had just been built. Its initial activities came out of a vision of 'community-based integrated care' [27,28]. Since then, the national health policy paradigm has shifted from centralized planning towards regulated competition [29,30] and the local population has changed dramatically, becoming more diverse and poorer [31,32]. As a result, we had the opportunity to describe and explore the complex relationship between the collaborative activities of the partnership, its nature and its changing context. For the description, in addition to the vision of community-based integrated care and the literature on the changing policy context, we used Janssen et al.'s theory on the nature of partnerships [33]. Their theory is based on six common organizational dimensions specified by the authors to describe partnerships (see Table 1).

We addressed the following research questions: 1) Has there been a community-based integrated care vision to the partnership's activities throughout the period of more than 30 years? 2) How did the nature of the partnership change over this period? 3) How did the partnership balance its nature and activities with the changing context over this period? In the discussion, we elaborate on the findings and draw lessons for adapting and sustaining a community-based integrated care vision in local health systems through health partnerships.

## Methods

A single case study design was considered the most appropriate to retrospectively explore the development of the partnership in South-eastern Amsterdam over time. Semi-structured interviews and document analysis were used to describe the nature of the partnership and its collaborative activities. Literature was reviewed to describe the transformation of the Dutch health policy context and the South-eastern Amsterdam community.

### Data collection

In 2003, we interviewed 17 key players selected on the basis of two sampling strategies (see Table 2). First, all managers (n = 11) currently representing member institutions were interviewed. Second, we used snowball sampling to identify key players who held leading and relatively independent positions in the past and could therefore have an overview of the development of the partnership during a specific time period. Respondents and documents were used to identify them (n = 6). Two researchers conducted face-to-face interviews with the respondents at their places of work using an interview guide, which was developed on the basis of the research questions (see Additional File 1). During interviewing, the guide was used in an informal and flexible manner. The interviews took approximately one hour each, were recorded and later transcribed. In addition, we collected documents. Former key players had preserved documents on the partnership in the 1970s and 1980s. Furthermore, we had access to an archive containing the partnership's documents dating back to 1989. Selected documents included officially published material such as annual reports, policy reports and research reports, and also working documents and meeting minutes (see Table 3). Finally, we did a concise literature review in order to find papers that reported on the Dutch policy context and the local community in South-eastern Amsterdam. An internet search was done for policy papers, including on the sites of the Ministry of Health, Sports and Welfare and the Municipal Health Service of Amsterdam [34,35]. Literature was also selected and gathered based on references made by respondents or in documents and/or literature.

### Data analysis

To answer the first research question, we analysed whether the collaborative activities manifested in the major projects of the partnership were initiated and developed based on the vision of community-based integrated care. This vision required that 1) projects be initiated and implemented based on prioritized community health needs; 2) projects be responsive to the beliefs, preferences and societal values of residents; and 3) projects be aimed at creating a seamless continuum of care – the implementation of mechanisms that would facilitate and coordinate service delivery at the right time and in the most appropriate setting [8]. In this way we distinguished between collaborative activities at the strategic level (e.g. the level at which strategic decision-making concerning resource allocation and investment takes place) and the operational level (e.g. the level at which service delivery is coordinated across people, functions and sites).

To answer the second question, we analysed documents and transcripts using the Janssen typology [33]. This typology includes six dimensions commonly used to describe organizations. The authors specify these to describe partnerships (see Table 3). They assume that independent organizations, which operate in a competitive context, can strategically use partnerships for innovation purposes. In this way, two innovative orientations can be chosen: a service-development orientation and an orientation based on improving existing services. The basic premise of the typology is that each orientation requires another way of organizing a partnership according to the six dimensions for effectiveness in achieving its goals. So, by describing the health partnership in Amsterdam over time according to these six dimensions, it was possible to detect transformations in the nature of the partnership. This typology was chosen because Dutch health-care providers must increasingly compete with each other and the partnership in Amsterdam has traditionally been used as an instrument for innovation.

To address the third question, we used an open approach to explore the balancing process between the partnership and its context. The focal points were the crises underlying the formation and transformations of the partnership. We expected that balancing processes would be most visible during these periods. Because of this, we took a 'systems perspective', as the literature shows that factors that influence the effectiveness and sustainability of health partnerships are highly interdependent and cannot easily be isolated from each other [26].

### Ensuring rigour

We used several strategies to monitor and enhance the rigour of the data as well as to rule out validity threats. First, we tried to cross-validate key findings by triangulation of data. The data collected from different sources (i.e. semi-structured interviews, document analysis and literature) were simultaneously analysed and reported. Second, we presented our preliminary findings to most of the respondents during a partnership meeting in December 2003. Furthermore, we sent Respondents 1 and 7 a draft of our manuscript and asked them to check the analysis and interpretations. Respondent 1 is the only one who has been involved with the partnership throughout the entire time period; Respondent 7 is the current chair and our principal contact. We used both ways of checking our analysis to validate the findings. Third, we solicited feedback from senior and other researchers (*peer review*), who critically appraised an earlier draft of the manuscript. Fourth, there were regular meetings of the co-authors throughout the entire study period. During these meetings, the data collection and analysis were monitored and discussed.

## Results

### The partnership's activities

The vision of community-based integrated care could be seen in the activities of the partnership, though it disappeared over time. This is illustrated by the partnership's engagement in 12 projects developed since 1973 (see Table 4). All 12 projects were targeted at the South-eastern Amsterdam community. However, a comparison of the first projects (1–5, 7) with the most recent ones (6, 8–12) showed a shift from serving the entire community towards serving the elderly and chronically ill. Moreover, the projects were not initiated on the basis of prioritized community health needs. Only the earliest projects (1–4) used demographic data (sometimes supplemented by epidemiological data) to plan the volume and capacity of care institutions that had to be built [b, d, g]. The projects in the later periods were supply-driven, as they were initiated on the basis of observed care gaps (projects 6, 8–11) or on opportunities that arose because of requests by the local sickness fund (projects 5, 7) or the changing policy context (12). Community health data were occasionally used (projects 8, 10, 12) to estimate the number of eligible patients in the community. Still, this information was used more to investigate the feasibility and profitability of potential integrated care arrangements rather than to prioritize and set up services to maximize population health.

A similar pattern was visible for the community links that were established. There was real participation by residents in the projects 1 through 4. This is best illustrated by the study on community participation in the primary care centre in Holendrecht [36]. In this centre, the community was systematically engaged by making community representatives members of the board, organizing bi-monthly discussion meetings concerning care services delivered and organizing an annual forum open to all community residents interested in the centre. Respondents (1–3), documents [b, d, g] and research [27,28] substantiated this finding. In the later projects (5–12), we were no longer able to find this kind of systematic involvement of the community.

The integration of activities at the strategic level diminished over time. It was the highest in the earlier projects (1–8), as strategic functions such as planning services, innovation, regional needs assessments, care commissioning, resource allocation and information management were centralized at the partnership level. This was done because member institutions could not initiate, develop and realize the projects themselves. Realizing successful projects required influence on national and local health policy-making for which joint strategic action was needed. In the later projects (9–12), strategic functions were more decentralized. Member institutions initiated and developed innovative projects themselves or in small coalitions. Projects were put on the agenda and discussed, but primarily to inform member institutions.

The integration at the operational level in the projects was initially modest, but later increased and stabilized. In the first projects (1–4), operational activities were predominately focused on creating the prerequisites for integrated health-care delivery. For example, the partnership arranged accommodation for the centres and set up meetings for exchanging experiences among professionals on how to set up a primary care centre [d]. However, the partnership was not engaged in standardizing the processes in terms of responsibilities, protocols, information transfer, monitoring and feedback mechanisms. Nor did it have a role in the actual health-care delivery to individual patients.

This changed in the later projects (5–11), in which the partnership executed various operational activities. For example, in the stroke service project (8), the partnership's employees chaired weekly steering committee meetings, assessed health-care needs, commissioned health care or mediated admission to an institution, monitored waiting lists in the participating institutions, registered all relevant information including transfers in an integrated patient record, communicated with patients and their carers, and could be consulted for advice [o, r]. These operational activities could also be identified in the other projects except for project 12 [x].

### The nature of the partnership

Based on differences in the typology of the partnership over time, three time periods were identified: the SGZ period (1973–1989), the Zizo I period (1989–1997), and the Zizo II period (1997-present). In the SGZ period, the focus of the partnership was on service development – that is, building a community-based integrated care system with its emphasis on primary care [a, b, d, g]. The governance of the partnership was focused on serving the collective interests of the participating organizations, which essentially implied the realization of primary care centres in the area. An example of this are the efforts to realize a primary care centre in a neighbourhood where there were already individual general practitioners (GPs). The partnership firmly supported this initiative, thereby ignoring the opposition of some GPs with individual practices who were also participating in the partnership [d]. The structure was flexible, informal and with no hierarchies. It featured an executive committee supported by a small self-supporting executive office that supervised, managed, facilitated and coordinated the partnership's activities [28]. There was a strong core culture based on the aforementioned vision and its underlying social-democratic standards and values. The strategic fit was high, because all relevant stakeholders in the community participated or were indirectly involved (see Table 5). Finally, the partnership management was dynamic and unregulated, emphasizing a long-term perspective and interactive approach.

In the Zizo I period (1989–1997) the partnership focused both on service development and improving the existing services. Innovations featured new ways of integrated care delivery (project 5) as well as streamlining existing care processes – that is, coordinating and aligning the input of all professionals and institutions involved (projects 6–8). Governance concentrated on balancing the collective interests with the individual interests of participants. As formulated in the minutes of committee discussions in 1990, transparency and clarity were considered essential to nurturing fruitful negotiations, compromises, and experiments [j]. In this regard, respondents (1, 4, 5, 8) also referred to a common remark used to express the unwritten rule of transparency:

"'Well understood own interests" was a comment we often used.' (Respondent 4)

The structure of the partnership was more formal and binding. The partnership's organization featured a two-person management team (i.e. a director and a secretary) and an executive office known as 'circuit management' that coordinated, executed and supported the collaborative activities. This organization was paid for in part by the participants themselves. The strategic fit changed as the public health office, community organizations and financiers (municipality of Amsterdam, sickness fund) withdrew from the partnership (see Table 5). Lastly, there was strong management of the partnership by the management team, who prepared the partnership meetings and headed up the executive office.

In the Zizo II period (1997-present) the focus was on innovative activities, which mostly elaborated on the expertise of the circuit managers and the existing infrastructure. This is illustrated by the most recent projects (9–12), which build on the expertise acquired during the earlier projects. Documents [n, p, q, s] and respondents (1, 4, 5, 6) substantiated this finding. Governance concentrated on seeking coalitions based on shared interests rather than on collective interests. This was explicitly stated: 'New projects are initiated by two or more health-care providers. One of these initiators is now responsible, not the Zizo.' [p]. Even so, the partnership culture was less dominant; each representative propagated the interests of their own organization rather than the collective ones.

'You are allowed to have your own interests. You don't have to hide them.' (Respondent 8)

The partnership structure was downsized by removing the management team. The circuit management (staffed by the three circuit managers) was maintained. Furthermore, an additional type of meeting was organized. What were known as 'health-care provider meetings' were held occasionally to prevent individual patients falling through the cracks [q, s]. The partnership management was minimal, because the executive committee only prepared the meetings and supported the self-directed circuit managers. Finally, the strategic fit between the member institutions weakened, because the institutions for care of the elderly increasingly offered similar services.

### The partnership in relation to its context

**Figure 1:**
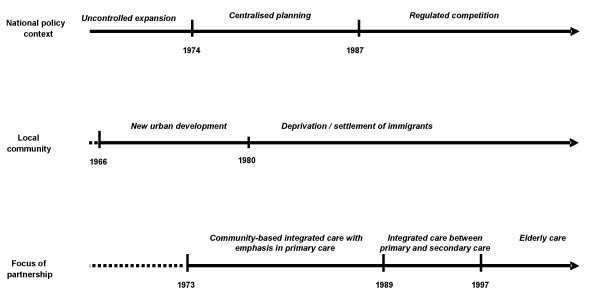
The focus of the partnership and its relationship to the local community and national policy context.

Figure 1 provides an overview of the partnership in relation to its context. The figure indicates that the identified transformations on these three dimensions relatively coincided, and thus influenced each other.

The formation of the partnership in the 1970s went well. The health-care infrastructure in South-eastern Amsterdam had to be built from scratch, and this offered unique opportunities for experiments and innovation. At that time, the segregated health-care system and its uncontrolled expansion were considered problematic [28]. This was acknowledged by a foundation known as the SCAB (Stichting Contact & Adviesorgaan Bijlmermeer, 'Bijlmermeer Contact & Advisory Body') and later the SOSB (Stichting Ontwikkeling Subcentra Bijlmer, 'Foundation for the Development of Bijlmer Sub-centres') [a]. This organization encouraged discussion and promoted new ways of public service delivery drawing on the concepts of 'community-based care' and 'integrated care' before these terms existed.

Its ideas became influential through the support of stakeholders such as the municipality of Amsterdam, the sickness funds in Amsterdam and local community organizations. Moreover, in the early 1970s the SCAB attracted young health-care professionals, brought together in the 'Primary Health Care Working Group' (Werkgroep Eerstelijnszorg). Respondents (1, 2) said that this group of people was willing to implement the SCAB's vision and work accordingly. The partnership became successful against this backdrop.

'Yes, the partnership [during the first period] has been very successful. (...) There was an integrated vision. There were people who wanted to work according to this vision. There was the power to implement this. Even so, the circumstances were good: an easy geographical area and the support of financiers.' (Respondent 3)

In the mid-1980s, the spirit dissipated for several reasons. First, respondents (3, 4) noticed that the partnership's mission had been accomplished with the successful establishment of the primary care centres and the new ambulatory mental health-care organization. Second, the planned urban development turned out to be disastrous [27,28]. Due to a mistaken philosophy and flawed implementation, the district deteriorated rapidly and to a considerable degree. In less than a decade, people from low socio-economic backgrounds and ethnic minorities settled here, while those who were more affluent left. With these demographic changes came societal problems such as unemployment, criminality and ethnic segregation. Within this context, community organizations lost their vitality, which was made even worse by the local and national governments, who severely limited their social care subsidies. First and foremost, the national health policy paradigm shifted from centralized planning towards deregulation and competition [29,30]. Maintaining partnerships like the SGZ did not fit in to this new paradigm. So, financiers such as the municipality of Amsterdam and the regional sickness fund stopped their subsidies and withdrew their membership, saying that the members had to fund the partnership themselves if they valued the collaborative activities so much. Consequently, the partnership found itself in crisis and with serious funding problems [27,28].

'The financing of these types of organizations was stopped. (...) We said to each other – especially the former director of the AMC – that if we want to extend our planning work towards future issues in health care and maintain the collaboration, we have to fund the partnership ourselves.' (Respondent 1)

The partnership continued at this crossroads, although it was radically transformed into a collaboration of health-care providers only. Visionary key players anticipated new opportunities for collaboration, as articulated in the concept of integrated care that was introduced at that time [37]. They convinced most others to continue the partnership, although within a more binding structure.

'Everyone needed – everyone? no, not everyone – a great many needed that kind of binding structure, in which parties were a bit more committed to each other than in the SGZ. That was also the spirit of the times. If you want to organize a chain of care, you can't do this based on noncommittal agreements.' (Respondent 5)

Political pressure was needed to keep the social care agencies in, as they were merging and reluctant to give away autonomy. For a similar reason, the home care agency timely suspended its membership. Representatives were discontented by the intensive home care project (project 5) that they considered to be their exclusive jurisdiction. The public health office and community organizations left for other reasons. The municipal health service Amsterdam simply left as a natural outgrowth of the withdraw of the municipality of Amsterdam. The community organizations were dropped out, because of their own declining vitality as well as the disinterest amongst the participating care providers.

' [The dropout of community organizations] went too easy. We did not think it over very well. The idea was, we cannot ask them to pay a contribution and the new norm was: he who pays the piper dictates the tune.' (Respondent 1)

Respondent 5 acknowledged this, but also underscored that there was no other option for the partnership. The core-business was to collaborate and set up integrated care arrangements among care providers. A systematic discussion with the community was not the primary aim anymore. Due to the dropout of all these organizations, the partnership got a health-care orientation with no link to public health and a weak one to social care.

After the final decision to continue the partnership in 1989, the partnership was converted into an association funded by contributions by the participating institutions themselves. Soon, funding for project 5 was realized. Respondents (1, 4, 5) emphasized the enormous spin-off of this project, which was also substantiated by documents [j-l]. First, the regional sickness fund contracted the partnership to control the entire budget of intensive home care in South-eastern Amsterdam, thus giving the partnership a strategic position in the local health system and showing its usefulness. Second, in order to adequately run this project, the partnership had to set up and organize an administrative bureaucracy. This circuit management was staffed by professionals who soon became the experts on integrated health-care arrangements at the operational level.

'We started with the intensive home care project, which in my view has been an important stimulus. (...) If you can show your member organizations it's profitable to collaborate on successful projects, then you start a motor that encourages collaboration in the broadest sense, also at the strategic level.' (Respondent 4)

'It gave us a tremendous opportunity, but our organization was totally unprepared. It meant that you had to control financial flows, that you needed to have an administrative system, and that you needed to have people to do the job.' (Respondent 5)

The expansion resulted in a partnership that had the expertise, the capacity and the support to initiate, develop and realize innovative and successful projects.

In the mid-1990s new tensions arose. The incremental shift towards regulated competition resulted in defensive organizational behaviour. To reduce uncertainty, various member institutions merged (especially the nursing homes and residential homes), becoming part of large enterprises. Furthermore, one ad hoc intervention by government torpedoed core project 7. Legislation imposed the installation of 'Regional Individual Needs Assessment Agencies' (RIOs), which had to be organized independently of health-care providers [38]. Lastly, the policies of the regional sickness fund were inconsistent, which made their support less effective.

These developments brought about a new crisis. First, the partnership's organization had to be dismantled. The staff that had been doing health-care needs assessments had to leave and/or work for the independent RIO in Amsterdam known as 'Tot & Met'. Because the operational activities were funded primarily on the basis of this project, the partnership ran out of financial resources. First and foremost, the crisis triggered a discussion on sustaining the partnership. The merged institutions had an orientation that exceeded the local health system, and they strategically aimed to offer seamless continuums of care services within their own institutional borders. Still, they remained committed to the partnership. By chance, all four merged institutions active in the entire Amsterdam area were represented in South-eastern Amsterdam, which inhibited the dominant market position of one of them (Respondent 15). Apart from that, they highly valued the work of the circuit managers, whose expertise and experience should be maintained and used for future innovations [k].

'The situation of the partnership was quite desperate. Why do we still have a ZIZO? (...) I thought, if we want to maintain the partnership, we have to keep the circuit managers operational.' (Respondent 6)

This ultimately resulted in a new vision that promoted the improvement of existing collaborative activities and capabilities in the domain of care for the elderly and the chronically ill [q, t].

## Discussion

Several European governments promote health-care partnerships as appropriate vehicles for dealing with the tension between collaboration and competition [14,15,20]. This tension is driven by two divergent trends. First, collaboration among health-care providers is considered necessary in order to meet the health needs of populations with chronic diseases and other typical morbidity patterns of the fourth stage of epidemiological transition [4-7]. Second, the experiments with regulated competition in many industrialized health systems trigger health-care providers to secure their own economic viability and optimize specific individual organizational goals rather than taking collective action to improve community health [9]. It is suggested that health partnerships can reconcile both trends [14,15,20].

The study presented in this paper challenges this view. However, due to the explorative and retrospective nature of our study, the data sources used and its contextual imperative, the transferability of the findings to other settings is limited. Still, we believe the findings have a broader meaning. First, validity threats were ruled out by the comprehensive data gathered and methodological approach followed. So, the study has provided a credible exploration of the development of a health-care partnership in Amsterdam over more than 30 years. Moreover, the long time horizon of the study was favourable: 1) It gave us the opportunity to explore the outcomes of a health-care partnership over time, as manifested in the projects; and 2) We could track the influence of competitive elements on the nature and activities of a health-care partnership. Given the existing knowledge gap and the political rhetoric concerning health partnerships [23], the findings therefore provide useful insights. These are articulated in the following notions:

First, the 30-year history of the South-eastern Amsterdam partnership shows that the partnership successfully realized 12 collaborative projects and was sustained by changing its strategic goals over time. Its mission shifted from shaping a community-based integrated health system with its emphasis on primary care, by way of substituting integrated care arrangements for hospital care, towards elaborating on successful integrated care arrangements in care for the elderly. However, with this shift the partnership's activities were no longer planned on the basis of prioritized community health needs, and the community links fell away. This finding casts doubt on health partnerships' ability to implement programmes on the basis of a community-based integrated care vision. It indicates that the link between population health and health service delivery cannot be made by a health partnership of competing health-care providers only. Governing bodies and financiers should encourage a population-health focus and ensure that partnerships not only do things right but also do the right things.

This brings us to the second notion: External forces had an unmistakable impact on the partnership and its activities. Known contextual factors from the literature (i.e. financial incentives, health policies, the physical environment, cultural climate) were all evident [21-25]. Their accumulated impact gradually confined the partnership in its activities. Initially, the room for discretion was quite large as community organizations, the municipality and the regional sickness funds were committed creating fertile ground for community-based integrated care. However, the incremental implementation of regulated competition and the demographic changes marginalized the role of the municipality, the public health office and the community organizations. Without their input, the partnership was unable to initiate needs-based projects.

Conversely, the role of the sickness fund increased. It became the most crucial stakeholder of the partnership. At first, this was beneficial. Later, the governance of the sickness fund was more ambiguous. This had to do with the increased financial risks concerning quality-based purchasing and the financial risks taken by the major health-care insurer in Amsterdam. This relates to the international debate on incorporating population-health considerations in health system governance. Governments currently delegate governance roles to competing health-care providers and/or financiers. However, incentives that trigger the uptake of a population-health perspective are often ignored [12,13]. Therefore, it is uncertain whether governance bodies will adapt a community-based integrated care vision.

Apart from influences of national policies and local governance practices, the development of the partnership was also the natural outgrowth of building a local health care system from the scratch. Lewis et al. (2000) argue that rearranging health care in favour of more preventive and social interventions rearranges resources and incomes, creates winners and losers in a finite world [9]. At the outset, there were no health-care providers with vested interests, who could become losers and oppose to identifying population health needs and involving the community organizations. This situation gradually changed with the accomplishment of the health-care infrastructure in South-eastern Amsterdam. So, this contextual factor adds up to the explanation why the partnership lost its 'community-based' character overtime.

Despite the confinement of the room for discretion by external forces, the partnership was able and flexible enough to adapt and to survive. There is a broad range of literature discussing the critical capabilities and competencies necessary for sustaining health-care partnerships. In this paper we do not pinpoint specific capabilities and competencies that were decisive in Amsterdam. Rather, from a more abstract level our analysis indicates that the major driving force underlying the sustainability of the partnership has been its formation in the first place. Once the partnership was institutionalized and operational, it kept on nurturing its own survival. The people involved in and employed by the partnership interchangeably celebrated the partnership itself or successful projects or both in order to survive. In these processes, known factors such as personal factors, leadership, trust and culture were indeed evident.

Taking these three basic notions together suggests that encouraging health-care providers to collaborate and compete at the same time will not lead towards a needs-based and integrated health system. Seamless continuums of care will be realized, but these will not necessarily maximize community health. Governing bodies have to govern partnerships through active policies related to health system design, incentive structures and population-based performance measurement in order to ensure that community-based integrated care is more than just policy rhetoric.

## Conclusion

Through partnerships, health-care providers can build seamless continuums of care. However, these will not automatically maximize community health within a context of limited resources. Active policies with regard to health system design, incentive structures and population-based performance measures are warranted to ensure that community-based integrated care through health partnerships is more than just policy rhetoric.

## Competing interests

The author(s) declare they have no competing interests.

## Authors' contributions

TP designed the study, conducted semi-structured interviews, analysed the data and primarily wrote the paper.

DMJD designed the entire study, reviewed earlier drafts of the paper and supervised the research activities.

NPCH conducted semi-structured interviews, helped analyse the data and critically reviewed earlier drafts of the paper.

NSK was responsible for completing and supervising the study, designed the entire study and critically reviewed earlier drafts of the paper.

## Pre-publication history

The pre-publication history for this paper can be accessed here:



## Supplementary Material

Additional File 1 Topic list. Click here for file

## Figures and Tables

**Table 1:** The six interrelated dimensions of the typology for describing the nature of the partnership over time.

	Dimension	Definition
Organizational characteristics	Strategic orientation	The extent to which the partnership has a strategic orientation towards innovation through new service development or improvement of existing services.
	Partnership governance	The extent to which the partnership's governance is strategically directed towards serving collective interests.
	Partnership culture	The extent to which standards and values are shared between the participating care organizations.
	Partnership structure	The extent to which collaborative activities are organized into formal structures.
Managerial characteristics	Strategic fit	The extent to which the participating health-care organizations fit together to achieve the partnership's goals.
	Partnership management	The extent to which management processes such as decision-making, negotiations and knowledge exchange are regulated.

Source: Janssen APJ, Volberda HW, Asselberg CM [33].

**Table 2:** Interviewed respondents and their involvement with the partnership.

No.	Position	Institution	Time period involved
1.	Former treasurer, SGZ Representative member institution	GP association, Amsterdam	1973 – present
2.	Former chair, SGZ	Independent	1973 – 1987
3.	Former project coordinator, SGZ	Independent	1979 – 1982
4.	Former secretary, Zizo	Independent	1989 – 1997
5.	Former director, Zizo	Independent	1989 – 1997
6.	Former interim director, Zizo	AMC/University of Amsterdam	1991 – 1998
7.	Chair, Zizo	AMC/University of Amsterdam	1998 – present
8.	Committee member, Zizo	Evean Care Amsterdam/Henriette Roland Holst Huis	1997 – present
9.	Treasurer, Zizo	Verenigde Amstelhuizen/Gaasperdam nursing home	1999 – present
10.	Representative, member institution	AMC/de Meren	1996 – present
11.	Representative, member institution	'Amsterdam Home Care'	2002 – present
12.	Representative, member institution	'Amsterdam Home Care'	2001 – present
13.	Representative, member institution	Verenigde Amstelhuizen/De Diem residential home	2002 – present
14.	Representative, member institution	'Amsterdam Care Group'/Nellestein residential home	2003 – present
15.	Former treasurer, Zizo Representative, member institution	Fontis/De Venser residential home	1989 – 2003
16.	Representative, member institution	MADI 'Social Care for South-eastern Amsterdam'/Diemen	2000 – present
17.	Representative, member institution	GAZO 'Association of Primary Care Centres'	2001 – present

**Table 3:** Selected documents.

a.	Stuurgroep Gezondheidszorg Zuidoostlob. 'Report of the steering committee on health care for South-eastern Amsterdam' [in Dutch]. Amsterdam: Stuurgroep Gezondheidszorg Zuidoostlob. 1973.
b.	Stichting Gezondheidszorg Zuidoostlob (SGZ). ' 1977–1978 Annual Report' [in Dutch]. Amsterdam: SGZ. 1979.
c.	Beheersgroep Bijlmermeer. Notedop 1979. Amsterdam: Beheersgroep Bijlmermeer. 1979.
d.	Stichting Gezondheidszorg Zuidoostlob (SGZ). '1979 Annual Report' [in Dutch]. Amsterdam: SGZ. 1980.
e.	Stichting Wijkbouworgaan Bijlmermeer (SWOB). Van de Bijlmer méér maken. Een deltaplan voor de Bijlmermeer. ('Make more of the Bijlmeer'. In Dutch.) Amsterdam: SWOB. 1980.
f.	Maesen, van der LJG 'Outpost outpatient clinics Ganzenhoef to change the relationship between the new academic hospital and its environment, the Bijlmermeer and surroundings' [in Dutch]. Amsterdam Boskoop: Somso/Macula. 1982.
g.	Stichting Gezondheidszorg Zuidoostlob (SGZ). '1980–1981 Annual Report' [in Dutch]. Amsterdam: SGZ. 1983.
h.	Zizo. 'Project description for home care in South-eastern Amsterdam for the period 1990–1992' [in Dutch]. September 1989.
i.	De Zuidooster. Newsletter for health care and social care delivery [in Dutch]. No. 1, 1990.
j.	Van der Mast, M. Minutes of the meeting concerning the Zizo [in Dutch]. 11-09-1990.
k.	Boekholdt, MG. Comments on the collaborative structure in the region Southeast [in Dutch]. Tilburg, March 1991.
l.	Zizo. 'Zizo 1992 Annual Report/evaluation project intensive home care 1990–1992' [in Dutch]. May 1993.
m.	Boekholdt, MG. 'The functioning and structure of the Zizo'. Recommendations [in Dutch]. Utrecht, September 1996.
n.	Mast van der M. 'Report on strategic exploration of the Zizo's future' [in Dutch]. September 1997.
o.	Zizo. Stroke service project, South-eastern Amsterdam/Diemen. '1997/1997 Annual Report'. December 1997.
p.	Zizo-board. 'How to continue the Zizo?' [in Dutch]. December 1997.
q.	Zizo. 'Profile at the Table' [in Dutch]. 21 April 1998.
r.	Academic Medical Center. 'Book of Protocols, Stroke Service Amsterdam. South-eastern & Diemen regions' [in Dutch]. June 1998.
s.	Schurink, R. 'The care provider table in South-eastern Amsterdam' [in Dutch]. Diemen, 29 September 1998.
t.	'Letter of intent, ZiZo association' [in Dutch]. Signed in April 2002.
u.	KOZ. 'Integrated care of the elderly in South-eastern Amsterdam and Diemen. Preliminary document' [in Dutch]. November 2002.
v.	Hoogedoorn, N. 'Project proposal for home care coordination. Revised version' [in Dutch]. December 2002.
w.	Zizo. 'Emergency psychogeriatric care, South-eastern Amsterdam and Diemen' [in Dutch]. November 2003.
x.	MaDi, Verenigde Amstelhuizen South-eastern region, Henriette Roland Holsthuis. 'Community care service points. Working plan' [in Dutch]. February 2004.

**Table 4:** Activities of the partnership as manifested in its major projects.

No.	Project	Aim	Community orientation	Partnership's involvement	Status
1.	'Bijlmer Social Care Centre*	To establish a community-based, decentralized, integrated social care institution to prevent fragmented and compartmentalized social care delivery.	The centre was established in the South-eastern Amsterdam district. Community intelligence was used to plan the volume and capacity of the centre. Residents actively participated in the development of the project.	At the strategic level, the partnership influenced local policy-making processes by promoting the establishment of the centre.	The centre was realized in 1972.
2.	Primary care centres	To establish decentralized and multifunctional primary care centres.	All centres were established in the South-eastern Amsterdam district. Community intelligence was collected and used to plan the centres. Residents actively participated in the development of the centres.	At the strategic level, the partnership influenced local policy-making processes by promoting the establishment of the primary care centres. At the operational level, the executive office supported the primary care professionals who had the lead in setting up the centres.	Five primary care centres have successfully been established between 1975 and 1981 and still exist today. In 2001, the centres merged into the GAZO.
3.	Outpost outpatient clinic Ganzenhoef	To establish an outpost for medical specialist care and to develop innovative relationships with primary care.	Residents living in South-eastern Amsterdam who needed medical specialist care could use the outpost.	At the strategic level, the partnership discussed and developed proposals to continue the outpost and to use it as a vehicle for innovation.	The outpost outpatient clinic was closed in 1983 because support from the AMC fell away.
4.	Regional ambulatory mental care institution South-east (RIAGG)	To establish one decentralized institution for ambulatory mental health care.	The RIAGG was established in the South-eastern Amsterdam district. Community intelligence was collected and used to plan the institution. Residents actively participated in its development.	At the strategic level, the partnership influenced local policy-making processes by promoting the establishment of one RIAGG. At the operational level, the executive office supported the working group that had to develop and implement the plan.	The RIAGG has been operational since 1980, though more centrally organized than originally planned. In 1997 the RIAGG merged with the AMC and the Frederik van Eeden Stichting to form AMC/de Meren.
5.	Intensive home care/respite care beds	To organize health care as close as possible to the living environment of patients by optimizing the use of available expertise. This implied intensifying home care and establishing respite care beds in residential homes.	All residents living in South-eastern Amsterdam could make use of intensive home care and/or respite care beds. Community intelligence on production data (e.g. waiting lists, bed occupancy rates, needs assessments conducted) are continuously registered and regularly reported to manage the project.	At the strategic level, the partnership initiated, developed and realized the project. In order to execute the project, the partnership set up the circuit management that had to develop, implement, monitor, coordinate, control the budget and manage the project.	Since 1990, intensive home care and respite care beds have been developed and are operational.
6.	Shared care of psychogeriatric patients	To optimize the diagnosis, treatment and care for psychogeriatric patients by strengthening primary care and improving the input of secondary and tertiary care professionals.	All psychogeriatric patients residing in South-eastern Amsterdam can use this shared care service. Community intelligence on demographics and on the existing psychogeriatric care delivery was used to legitimize and manage the project.	At the strategic level, the partnership initiated, developed and realized the project. At the operational level, the circuit management developed, implemented, monitored, coordinated, and managed the shared care arrangement.	In 1989, a working group started to develop a project proposal. In 1992, a pilot was started. Since 1995, the shared care arrangement for psychogeriatric patients has been operational.
7.	Integrated care needs assessments	To assess care needs of patients and to allocate and initiate appropriate care.	The partnership was responsible for assessing care needs of all patients residing in South-eastern Amsterdam. Community intelligence on production data was registered and monitored and regularly reported to manage the project.	At the strategic level, the partnership the partnership initiated, developed and realized the project. At the operational level, the circuit management was responsible for conducting, registering and monitoring the integrated care needs assessments.	The project was operational between 1993 and 1997.
8.	Stroke service	To establish a complete, reliable and standardized supply of diagnosis, treatment, care, nursing and rehabilitation for stroke patients.	All stroke patients residing in South-eastern Amsterdam are eligible for admission. Community intelligence on the prevalence of stroke was used to estimate the number of patients a year and production data are continuously collected to monitor and manage the service.	At the strategic level, the partnership developed and realized the project, which was initiated and developed primarily by the AMC. At the operational level, the circuit management coordinates, controls, monitors and registers all individual patients in the stroke service according to standardized criteria and procedures.	The project has been operational since 1996. In April 1998, the stroke service became a structural, integrated care arrangement in the region.
9.	Professional knowledge centre for care of the elderly	To develop a regional knowledge centre aimed at developing integrated care arrangements and care coordination for the frail elderly.	The project is targeted at all frail elderly residing in South-eastern Amsterdam.	At the strategic level, the partnership was involved in the initiation, development and realization of the knowledge centre.	The professional knowledge centre has been operational since November 2002.
10.	Emergency psychogeriatric care unit	To retain four nursing home beds for temporal and emergency admissions of psychogeriatric patients.	Only patients residing in South-eastern Amsterdam are eligible for admission. The unit was legitimized on the basis of monitoring data that showed inappropriate care for 10 to 15 patients a year in the community.	At the strategic level, the partnership was involved in the initiation, development and realization of the unit. At the operational level, the circuit management collects, assesses and registers applications according to standardized criteria and procedures.	The unit has been operational since January 2004 and four beds are available.
11.	Coordination of complex patients at home	To coordinate, align and supervise multidisciplinary care for complex patients by one assigned professional.	All patients with multidimensional health problems and living in the South-eastern Amsterdam are eligible for care coordination	At the strategic level, the partnership initiated and developed the service. At the operational level, the circuit management initiated and developed the service; it also supports the service on a day-to-day basis.	The one-year pilot has been evaluated. The service has now become structural.
12.	Community care service points	To support elderly residents living at home to find an appropriate mix of health care, social care and housing that meet their needs and wishes.	Nine service points are planned in the elderly care institutions located in South-eastern Amsterdam. Community intelligence on demographics and the local health system was used to legitimize the service points.	At the strategic level, the partnership got involved to approve, support, align activities and monitor the development of the service points.	The first three community service points became operational on 1 May 2005. The other six will be implemented at a later stage.

* This project was realized before the partnership was established.

GAZO: Gezondheidscentra Amsterdam Zuidoost (Primary health care centres South-eastern) Amsterdam.

AMC: Academic Medical Centre

RIAGG: Regionale Instelling Ambulante Geestelijke Gezondheidszorg (Regional ambulatory mental care institution)

**Table 5:** Member institutions of the partnership in the three periods.

Period Member institutions	1973–1989	1989–1997	1997-present
Public Health Office	Municipal Health Service Amsterdam		
Primary care centres	Holendrecht Gein Reigersbos Venserpolder Nellestein	Holendrecht Gein Reigersbos Venserpolder Nellestein	GAZO Primary care centres South-eastern Amsterdam
Professional associations	'Amsterdam Association of General Practitioners'	'Amsterdam Association of General Practitioners'	'Amsterdam Association of General Practitioners'
	'Amsterdam Association of Medical Specialists'		
Home care organizations	'Association of Collaborating Home Care Agencies in Amsterdam' Home care association for Diemen/Ouder Amstel	'Amsterdam Home Care'	'Amsterdam Home Care'
Social care agencies	Societal Center Bijlmer department of family care Societaql Center Bijlmer department of social care		MADI South-eastern Amsterdam & Diemen
Nursing homes & residential homes		Nursing home, Gaasperdam Home for assisted living, De Drecht Residential home, Eben Haëzer Residential home, De Diem Residential home, Onze Woning Residential home, De Venser Residential home, Nellestein Residential home, Henriette Roland Holsthuis	Verenigde Amstelhuizen Fontis Care group Amsterdam Evean Care Amsterdam
Hospitals	Wilhelmina Gasthuis Binnengasthuis	Academic Medical Center	Academic Medical Center/University of Amsterdam
University of Amsterdam	Faculty of Medicine (Academic Hospital University of Amsterdam)	Faculty of Medicine (AZUA)	
Mental health care	'Association of General Mental Health Care for South-eastern Amsterdam'	RIAGG (Regional Ambulatory Mental Health-care Agency)	Academic Medical Center/De Meren
Community organizations	SWOB association of community centres Bijlmer Driemond community organization Duivendrecht community organization Diemen association of community centres		
Financiers	'Association of Sickness Funds in Amsterdam' 'Haarlemmermeer Central sickness fund' Municipality of Amsterdam*		
