# Supplementary material for: Collaborating while competing? The sustainability of community-based integrated care initiatives through a health partnership
Source: BMC Health Serv Res. 2006 Mar 20;6:37. doi: 10.1186/1472-6963-6-37 (PMC1464130; doi:10.1186/1472-6963-6-37)
Supplement: Additional File 1 — Topic list. [file 1472-6963-6-37-S1.doc]

General objectives

- To explore the respondents perceptions and notions on the historical development of the partnership.
- To explore how the partnership have been dealing with contextual changes.

Introduction

- Introduce researchers and study; confidentiality; research procedure

Personal characteristics and background

- Sure name, sex, function
- Background, experience with the partnership, education

Openings question: Can you tell me something about your (past) involvement in the partnership?

- Role and function
- The acquaintance / expectations
- Experiences / anecdotes

Question: What strategic goals did the organisations strived for by participating in the partnership?

- Did these goals change over time?
- Convergent or divergent goals / long term of short term
- type of collaboration
- collective or individual interests
- to what extent are these goals personally bound?

Question: If you had to distinguish different time periods. What periods would you identify?

- Why?
- SGZ / ZIZO old style / ZIZO new style?
- On what empirical evidence / illustrative discussions do you base this distinction?

Question: Can you typify the partnership in each time period?

- Strategic fit (Partnership composition)
- Structure
- Culture (norms & values, subcultures, core culture, socialisation processes)
- Funding

Question: To what extent is the development of the partnership influences by external factors (environment/context)?

- What factors (National policy, local policy, demographics, epidemiology)
- Merging care providers
- Role of financiers / external stakeholders
- Deprivation of Amsterdam Southeast
- Does the partnership collect, exchange and use community intelligence in order to optimise and rationalise its decision making processes?\

Question: how did decision making processes of the partnership take place over time?

- Changed over time?
- Decisions based on consensus or majority?
- Extent of authority
- Allocation of responsibilities and authority to the partnership
- Dominance of one or more member institutions

End

- Ask how the respondent experienced the interview.
- Ask for feedback.
